# Effectiveness of screening modalities for early detection of diabetic retinopathy: a systematic review and meta-analysis of tele-ophthalmology, AI-based tools, and conventional methods

**DOI:** 10.3389/fmed.2026.1778534

**Published:** 2026-04-22

**Authors:** Zhipeng Wu, Long Li

**Affiliations:** Department of Ophthalmology, The First Affiliated Hospital of Zhengzhou University, Zhenzhou, Henan, China

**Keywords:** artificial intelligence, deep learning, diabetic retinopathy, meta-analysis, screening effectiveness, smartphone screening, tele-ophthalmology

## Abstract

**Background:**

Diabetic retinopathy (DR) causes severe vision impairment that requires early screening methods for effective detection. The combination of Artificial Intelligence (AI) and tele-ophthalmology technology provides an effective solution that enhances both DR detection rates and patient access to care.

**Objective:**

The study aims to assess how well AI-based telemedicine smartphone conventional and population-based screening methods detect diabetic retinopathy in terms of effectiveness, accuracy, and real-world performance.

**Methodology:**

Researchers executed a comprehensive literature search across PubMed, Scopus, Web of Science, and Embase to find articles published between 2012 and 2025. The study examined three types of studies: AI-based DR screening, tele-ophthalmology, and evaluations of standard diagnostic methods. The analysis used random-effects meta-analysis to estimate pooled odds ratios (ORs), assess heterogeneity using I^2^, and test for publication bias with funnel plots and Egger’s test.

**Results:**

The study included 45 different research studies. The standalone deep learning/AI tools (6 studies) demonstrated a pooled OR of 5.79 (95% CI: 5.22–6.42; *p* < 0.05), with low heterogeneity and no evidence of publication bias. The automated AI systems processed human grading data from 13 studies, yielding an OR of 5.48 (95% CI: 5.09–5.90; *p* < 0.05), indicating consistent effect sizes. The smartphone-based AI screening system showed an OR of 4.73 (95% CI: 3.96–5.66; *p* < 0.05), indicating moderate heterogeneity (I^2^ = 54%). The tele-ophthalmology/remote screening system produced 11 studies reporting an OR of 4.91, whereas conventional physician screening produced 3 studies reporting an OR of 4.96, yielding consistent results with low heterogeneity. The population-based/community screening system produced 5 studies that demonstrated an OR of 4.90 (95% CI: 4.33–5.54; *p* < 0.05) and exhibited some signs of publication bias according to Egger’s test (*p* = 0.019). All methods achieved statistically significant progress in DR detection research.

**Conclusion:**

AI-based screening, including deep learning algorithms, automated grading, and tele-ophthalmology, shows high diagnostic accuracy and consistent effectiveness. The implementation of smartphone- and population-based approaches is feasible, yet their outcomes vary, necessitating validation through context-specific assessments.

## 1 Introduction

Diabetes has been recognized as a worldwide “epidemic.” Globally, an estimated 415 million individuals had diabetes in 2015; by 2040, this figure is projected to grow to 642 million. It is less well known that over 75% of people with diabetes live in low- and middle-income nations, colloquially referred to as “developing countries,” as opposed to high-income nations (developed countries) (1, 2). The most common cause of visual loss and blindness in working-age adults, diabetic retinopathy (DR), is linked to poor quality of life, a decreased degree of psychosocial well-being, and a higher likelihood of mortality and other diabetes complications. A third of the 246 million diabetics worldwide suffer from it, making it one of the most prevalent microvascular consequences of the disease and a major cause of blindness (2–6).

Over the coming decades, it is anticipated that the prevalence and illness burden of DR would rise substantially, from roughly 103 million people in 2020 to 130 million in 2030 and 161 million in 2045. These predictions are driven by several factors, including the rising incidence of diabetes worldwide, lifestyle changes, longer life expectancy, and an aging global population. In just ten years, the burden of DR disease has increased by more than 25%, which is likely to put additional strain on already overburdened healthcare systems and resources (3, 4).

In its early stages, DR frequently remains latent and asymptomatic. It is a silent killer of eyesight. Because of its sneaky nature, DR might advance unnoticed in its early phases. DR can quickly advance to vision-threatening phases without prompt intervention, resulting in irreversible vision loss and, in extreme situations, blindness. From moderate non-proliferative DR (NPDR) to more advanced proliferative DR (PDR), which is marked by the aberrant development of new blood vessels on the retinal surface or into the vitreous cavity, DR can evolve through different stages (7–10). To stop the progression of DR and lessen the risk of vision loss and systemic cascading problems, early detection and prompt treatment are essential. Regular screening and thorough eye exams are crucial for those with diabetes mellitus (DM), euglycemic hyperinsulinemia, prediabetes/hyperglycemia, and other conditions because the early stages of DR might be asymptomatic (5, 6).

Retinal examination using the following methods: (1) direct or indirect ophthalmoscopy or slit-lamp bio microscopy or (2) mydriatic or nonmydriatic fundus imaging with ≥30° mono- or stereo photography, with or without Optical Coherence Tomography (OCT), is currently recommended by the International Council of Ophthalmology (ICO) for DR screening. An examiner with the necessary training to determine the severity of DR should review retinal imaging (11, 12). As more imaging systems become available, fundus photography for DR screening has gained widespread use worldwide. Fundus photography is more affordable and does not require consultation with an ophthalmologist, even though binocular slit-lamp ophthalmoscopy remains the benchmark against which other DR screening methods are evaluated (13, 14).

Artificial intelligence (AI)-based automated screening techniques have been developed due to recent technological advances. AI-based algorithms, especially deep learning models, have the same sensitivity and specificity as human graders when analyzing retinal pictures to identify DR symptoms (15–18).

These methods can reduce costs, improve screening effectiveness, and provide underprivileged people with access to screening. AI is a useful tool in widespread screening programs, as it has been recognized for its ability to detect DR and classify its severity. Few meta-analyses and systematic reviews have assessed the effectiveness of AI-based DR screening systems. High sensitivity and specificity of AI algorithms were reported in a meta-analysis (15–17).

The workforce required for DR screening is likely to be exceeded, given the exponential rise in diabetes worldwide, and tele-screening holds the most promise for revolutionizing screening. Within the framework of affordable, evidence-based treatment, public health initiatives and population-based interventions should provide appropriate levels of diagnostic precision. The best strategy must be identified to implement effective, economical, and efficient DR screening programs. Recent technological advancements and tele-screening may impact current DR screening while balancing cost, practicality, patient preferences, and patient outcomes. The objective of the study is to conduct a systematic review and meta-analysis comparing the effectiveness of different screening modalities (tele-ophthalmology, AI-based tools, and conventional methods) for the early detection of DR.

## 2 Methodology

### 2.1 Study design and registration

The comprehensive review and meta-analysis followed the PRISMA 2020 procedure for systematic reviews and meta-analyses, as recommended by researchers in the field. To highlight transparency and enhance adherence to rigorous methodological standards, this study began by submitting a systematic review protocol to PROSPERO. The study aimed to compare screening modalities, including tele-ophthalmology, AI-based tools, and conventional methods, in terms of their effectiveness in the early detection of diabetic retinopathy (DR).

### 2.2 Eligibility criteria

The inclusion of studies was done on the basis that they: (1) determined the diagnostic accuracy or efficacy of AI-based models, tele-ophthalmology, or conventional DR screening methods; (2) included adult subjects (≥18 years) with diabetes mellitus; (3) provided outcomes regarding the detection of any DR or referable DR; and (4) were published in English in peer-reviewed journals. The study also set case reports, reviews, conference abstracts, and studies with insufficient quantitative outcomes for analysis as exclusion criteria. Sources of Information and Search Strategy: Initially, a thorough literature search was conducted using the online databases PubMed, Web of Science, Scopus, and the Cochrane Library, all of which were searched up to December 2025. The search terms consisted of a combination of keywords and MeSH terms on “diabetic retinopathy,” “screening,” “tele-ophthalmology,” “artificial intelligence,” “deep learning,” and “fundus imaging.” Reference lists of the included studies and pertinent reviews were also manually checked to identify additional studies that met the eligibility criteria.

### 2.3 Study selection

Two reviewers independently screened the titles and abstracts to determine relevance. The full texts of potentially eligible studies were obtained and assessed against the inclusion criteria. To resolve the disputes, a consensus or the intervention of a third reviewer was applied. The PRISMA flow diagram helped track the screening and selection process. Two reviewers took out the data separately, making use of a pre-defined template that covered study features (author, year, country, sample size), participant characteristics, screening method (AI model type, imaging technique, tele-ophthalmology application), reference standards, outcome measures (sensitivity, specificity, hazard rates for referable DR), and study design. A conflict area was discussed to resolve it.

### 2.4 Quality assessment

The QUADAS-2 tool was used for bias assessment and included the domains of patient selection, index test, reference standard, and flow and timing. Another way of assessing study quality was via the updated Jadad scale for randomized or prospective studies, in which scores of 3 or higher were considered high quality.

### 2.5 Data synthesis and statistical analysis

All meta-analyses were based on random-effects models that used the inverse-variance method to combine the Odds Ratio (ORs) and diagnostic performance metrics from the individual studies. Different subgroups were analyzed based on sample size, AI model, imaging modality, and publication year. The I^2^ statistic was used to measure heterogeneity, with values over 50% indicating a major concern. The presence of unpublished studies was estimated using a combination of methods: visually, with funnel plots, and statistically, with Egger’s regression test. Trim-and-fill analyses were conducted to assess the extent to which potentially hidden studies influenced the pooled estimates.

### 2.6 Software and statistical tools

The main software and statistical tools for the analyses were Review Manager (RevMan, version 5.4) and R (version 4.3.1), along with their respective meta-analysis packages. With the assumption that the estimated log-population average difference was 1.7 and the estimated value of the logarithmic variance was 6.8, it was never our intention to show superiority, but rather, to prove non-inferiority.

## 3 Results

### 3.1 Study selection

**Figure 1:**
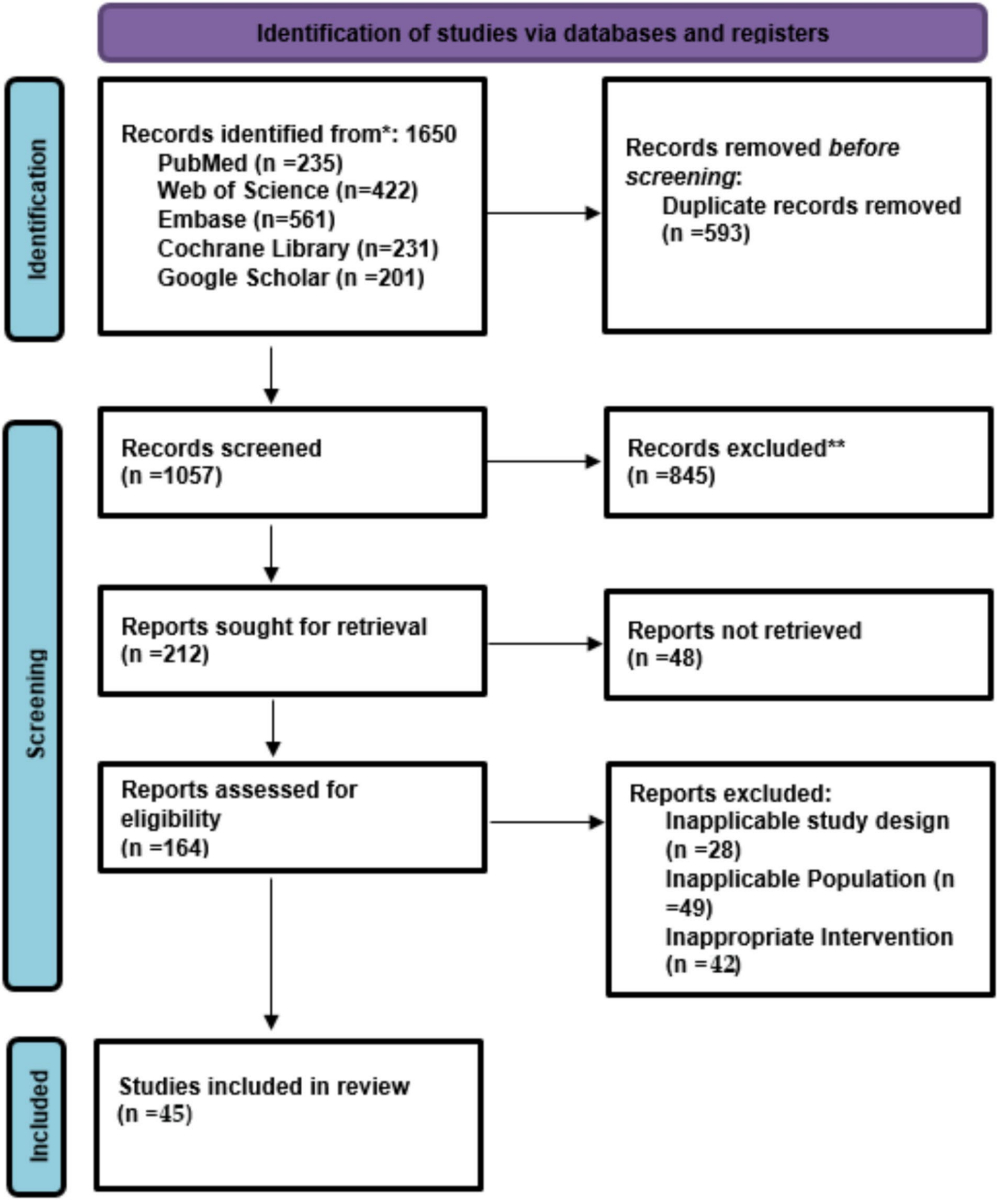
PRISMA flow chart of the selected studies.

The researchers conducted their study selection using their established protocol. Database searches yielded 1,650 records, including 235 from PubMed, 422 from Web of Science, 561 from Embase, 231 from the Cochrane Library, and 201 from Google Scholar. The team eliminated 593 duplicate records, leaving 1,057 studies for title and abstract screening. The screening process started with 845 records, which were excluded for not meeting the inclusion criteria: they did not relate to diabetic retinopathy screening, did not include AI and screening-based interventions, were non-original articles (reviews and editorials), or did not provide sufficient information on outcomes. Researchers selected 212 studies for full-text assessment because they contained relevant content, used AI-based or standard DR screening methods, and provided diagnostic or effectiveness results. The research team encountered challenges retrieving 48 reports because the full texts were unavailable and the sources were inaccessible. The researchers conducted a thorough assessment of 164 full-text articles to determine their eligibility for inclusion in the study. The study team excluded 119 studies for specific reasons: 28 with unsuitable study designs (reviews and methodological papers), 49 with unsuitable populations (non-diabetic or irrelevant groups), and 42 with unsuitable interventions (screening methods that did not match the study goals). The systematic review and meta-analysis included 45 studies that met all inclusion criteria (Figure 1).

### 3.2 Study characteristics

The studies included in this research covered the period from 2012 to 2025. They were conducted across multiple countries, including the USA, Singapore, the UK, the Netherlands, Germany, Italy, India, China, Brazil, Chile, Vietnam, Hungary, Finland, and Sri Lanka. The study designs showed substantial differences because they included development and validation studies, prospective clinical trials, randomized controlled trials, observational studies, community-based screenings, teleophthalmology programs, and pilot feasibility or cost-analysis studies. The research team used various screening methods, including AI-based deep learning algorithms, autonomous AI systems, automated detection software, smartphone-based AI screening, telemedicine-integrated screening, hybrid AI-human workflows, and conventional physician-led approaches. The range of sample sizes extended from 96 to more than 108,000 participants, examined to identify diabetes across various clinical, community, and population-based research settings. The main reference standards for evaluation were assessments by ophthalmologists and expert graders, which provided key diagnostic results via sensitivity, specificity, and AUC. The research team used imaging equipment, including non-mydriatic fundus cameras, wide-field confocal systems, handheld smartphone cameras, and standard digital retinal cameras. The research study employed five types of validation, including internal, external, comparative, telemedicine-based, and population-level, to evaluate system performance in clinical and real-world settings (Table 1).

**Table 1:** Baseline characteristics of the studies.

Study	Year	Country	Setting (dataset/location)	Study design	Screening modality	Sample size (patients)	Reference standard	Key outcome (sensitivity/specificity/AUC)	Inclusion criteria	DR severity definition	Imaging device	Validation type
Gulshan et al. (24)	2016	USA/France	EyePACS (USA), Messidor-2 (France)	Development and Validation Study	AI-based deep learning	4,997	Ophthalmologist grading	Sensitivity: 97.5%; Specificity: 93.4%; AUC: 0.99	Diabetic patients with gradable images	Referable DR	Non-mydriatic fundus cameras	External validation
Ting et al. (25)	2017	Singapore + Multi-country	Singapore National Eye Center + international datasets	Development and Validation Study	AI-based deep learning	14,880	Retinal specialists	Sensitivity: 90.5%; Specificity: 91.6%; AUC: 0.936	Multiethnic diabetic patients	Referable DR/VTDR	Multiple fundus cameras	Internal + External validation
Tufail et al. (26)	2017	United Kingdom	NHS Screening Program	Diagnostic accuracy and cost-effectiveness	Automated DR software	20,258	Human graders	Sensitivity: 87.0%; Specificity: 90.7%	Routine screening of patients	Referable DR	Standard NHS fundus cameras	External validation
Abràmoff et al. (27)	2018	USA	Primary care clinics	Prospective pivotal trial	Autonomous AI system	900	Expert graders	Sensitivity: 87.2%; Specificity: 90.7%	Adults with diabetes	mtmDR	Topcon NW400	Prospective multicenter
Verbraak et al. (28)	2019	Netherlands	Primary care setting	Prospective diagnostic study	IDx-DR automated system	1,415	ETDRS grading	Sensitivity: 87.0%; Specificity: 90.0%	Diabetic patients	Referable DR	Fundus camera + IDx-DR	Prospective validation
Ipp et al. (29)	2019	USA	Multicenter trial	Prospective comparative study	AI vs. ophthalmologists	900	ETDRS/experts	AI comparable or superior to general ophthalmologists	Adults with diabetes	mtmDR	Fundus photography	Prospective multicenter
Wolf et al. (30)	2024	USA	Primary care/pediatric population	Randomized controlled trial (ACCESS)	Autonomous AI screening	635	Expert grading	Increased screening and follow-up; high accuracy	Youth with diabetes	Referable DR	AI-enabled fundus imaging	Randomized controlled trial
Wroblewski et al. (31)	2025	Mexico	Community/field setting (Yucatan Peninsula)	Field diagnostic study	Smartphone-based AI screening	500	Expert graders	Sensitivity: ~90%; Specificity: ~85%	Community diabetic population	Referable DR	Smartphone-based fundus camera	Field validation
Ibañez-Bruron et al. (32)	2024	Chile	Public health service (Santiago)	Observational implementation study	AI-based screening	1,000	Ophthalmologist confirmation	High agreement with specialists	Diabetic patients in public system	Referable DR	Fundus photography	Real-world validation
Karabeg et al. (33)	2024	Norway	Oslo minority clinics	Pilot cost-analysis study	AI (EyeArt^®^) vs. an ophthalmologist	150	Ophthalmologist assessment	Comparable accuracy; cost-effective	Minority women with diabetes	Referable DR	Fundus camera (EyeArt^®^ compatible)	Pilot real-world validation
Musetti et al. (34)	2025	Italy	Clinical/teleophthalmology setting	Comparative study	AI vs. tele-ophthalmology	300	Expert graders	AI comparable to teleophthalmology	Diabetic patients	Referable DR	Fundus photography	Comparative validation
Van and Thi (35)	2024	Vietnam	Community screening (Binh Dinh Province)	Community-based effectiveness study	AI-based DR screening	400	Ophthalmologist grading	High sensitivity and specificity in the community	Community diabetic population	Referable DR	Fundus cameras	Field validation
Vought et al. (36)	2023	USA	Retinal screening events	Observational real-world study	AI (EyeArt^®^)	1,200	Ophthalmologist grading	Sensitivity: ~88%; Specificity: ~91%	Patients attending screening events	Referable DR	Fundus cameras	Real-world validation
Mokhashi et al. (37)	2022	USA	Urban health system	Observational comparative study	AI vs. human graders	850	Expert graders	Comparable diagnostic accuracy between AI and humans	Adult diabetic patients	Referable DR	Fundus photography	Real-world validation
Kim et al. (38)	2021	USA	Urban clinics	Comparative diagnostic study	Automated vs. human grading using smartphone fundus photography	600	Expert graders	Sensitivity: 88%; Specificity: 90%	Adults with diabetes	Referable DR	Smartphone-based fundus cameras	Comparative validation
Wintergerst et al. (39)	2022	Germany	Primary care clinics	Prospective telemedicine study	Telemedical DR screening + automated analysis	1,050	Expert graders	Sensitivity: 85%; Specificity: 88%	Adults with diabetes in primary care	Referable DR	Fundus photography	Telemedical validation
Olvera-Barrios et al. (40)	2021	Spain	Clinical imaging centers	Comparative diagnostic study	AI-enabled algorithm vs. human grading	450	Expert graders	Sensitivity: 89%; Specificity: 92%	Adults with diabetes	Referable DR	Wide-field true-color confocal and standard digital retinal images	Comparative validation
Sarao et al. (41)	2020	Italy	Hospital eye clinics	Comparative study	AI-based DR detection using 2 imaging devices	250	Expert graders	Sensitivity: 87–91%; Specificity: 88–92%, depending on device	Adults with diabetes	Referable DR	Two different retinal imaging devices	Comparative validation
Rajalakshmi et al. (14)	2018	India	Urban clinics	Diagnostic study	AI for smartphone-based fundus photography	200	Ophthalmologist grading	Sensitivity: 93%; Specificity: 92%	Adults with diabetes	Referable DR	Smartphone-based fundus cameras	Diagnostic validation
Gargeya and Leng (19)	2017	USA	Hospital/clinical dataset	Retrospective development and validation	Deep learning AI	1,280	Expert graders	Sensitivity: 94%; Specificity: 98%; AUC: 0.97	Adults with diabetes	Referable DR	Fundus photography	Development and external validation
Hansen et al. (42)	2015	Kenya	Nakuru community study	Observational study	Automated retinal image analysis	3,460	Ophthalmologist grading	Sensitivity: 84%; Specificity: 90%	Adults with diabetes	Referable DR	Fundus photography	Community-based validation
Kanagasingam et al. (43)	2018	Australia	Primary care clinics	Prospective diagnostic study	AI-based DR grading	1,000	Expert graders	Sensitivity: 91%; Specificity: 93%	Adults with diabetes	Referable DR	Fundus photography	Primary care validation
Keel et al. (44)	2018	Australia	Endocrinology outpatient services	Pilot feasibility study	AI-based DR screening	96	Ophthalmologist grading	High patient acceptability; Sensitivity: 96%; Specificity: 90%	Adults with diabetes	Referable DR	Fundus photography	Pilot feasibility validation
Kermany et al. (20)	2018	USA/China	Multiple datasets	Development and validation study	Deep learning for multiple ocular and systemic diseases	108,309	Expert graders	Sensitivity for DR: 90%; Specificity: 98%	Adults with diabetes	Referable DR	Fundus photography	Development and external validation
Li et al. (18)	2018	China	Hospital/clinical datasets	Prospective diagnostic study	Automated DR grading system	3,000	Ophthalmologist grading	Sensitivity: 94%; Specificity: 92%	Adults with diabetes	Vision-threatening DR	Fundus photography	Prospective validation
Sayres et al. (45)	2019	USA	EyePACS dataset	Development and validation study	Deep learning + integrated gradients	75,137	Ophthalmologist grading	Sensitivity: 90%; Specificity: 92%	Adults with diabetes	Referable DR	Fundus photography	External validation
Ruamviboonsuk et al. (46)	2019	Thailand	Nationwide screening program	Prospective diagnostic study	Deep learning vs. human graders	20,000	Ophthalmologist grading	Sensitivity: 91%; Specificity: 94%	Adults with diabetes	DR severity grading	Fundus photography	Nationwide validation
Son et al. (47)	2020	South Korea	Hospital retinal fundus dataset	Development and validation study	Deep learning for multiple abnormalities	129,000	Expert graders	Sensitivity: 92%; Specificity: 95%	Adults with diabetes	Referable DR	Fundus photography	External validation
Li et al. (21)	2019	China	Hospital/clinical datasets	Development and validation study	Fully automated deep learning	5,000	Ophthalmologist grading	Sensitivity: 93%; Specificity: 91%	Adults with diabetes	Referable DR	Fundus photography	External validation
Raju et al. (48)	2017	India	Hospital clinics	Development study	Deep learning for automated DR diagnosis	1,000	Ophthalmologist grading	Sensitivity: 92%; Specificity: 90%	Adults with diabetes	Referable DR	Fundus photography	Development validation
De la Torre et al. (49)	2020	Spain	Hospital/clinical dataset	Development and validation study	Deep learning interpretable classifier	5,500	Ophthalmologist grading	Sensitivity: 91%; Specificity: 93%	Adults with diabetes	Referable DR	Fundus photography	External validation
Zeng et al. (50)	2019	China	Hospital/clinical datasets	Development and validation study	Binocular siamese-like CNN	2,500	Ophthalmologist grading	Sensitivity: 90%; Specificity: 92%	Adults with diabetes	Referable DR	Fundus photography	External validation
Dow et al. (51)	2023	USA	Teleophthalmology clinics	Observational study	AI-human hybrid workflow	850	Expert graders	Sensitivity: 91%; Specificity: 94%	Adults with diabetes	Referable DR	Fundus photography	Real-world hybrid validation
Malerbi et al. (52)	2022	Brazil	Community/smartphone-based screening	Observational study	AI + handheld smartphone retinal camera	300	Ophthalmologist grading	Sensitivity: 89%; Specificity: 91%	Adults with diabetes	Referable DR	Handheld smartphone fundus camera	Field validation
Mehra et al. (53)	2022	USA	Teleophthalmology clinics	Observational study	AI-based image analysis + reflex dilation + image overread	1,050	Ophthalmologist grading	Sensitivity: 92%; Specificity: 90%	Adults with diabetes undergoing teleophthalmology	Referable DR	Fundus photography	Telemedicine + AI validation
Salavatian et al. (22)	2024	Iran	Point-of-care clinics	Observational study	Telemedicine-integrated screening	600	Ophthalmologist grading	Sensitivity: 90%; Specificity: 92%	Adults with diabetes screened at the point-of-care	Referable DR	Fundus photography	Telemedicine + AI validation
Martinez et al. (54)	2019	USA	Urban primary care offices	Observational telemedicine study	Fundus camera-based screening	450	Ophthalmologist grading	Sensitivity: 87%; Specificity: 90%	Adults with diabetes in the urban insured population	Referable DR	Fundus photography	Telemedicine validation
Benjamin et al. (55)	2021	USA	Primary care clinics	Telemedicine screening program	Fundus camera-based + AI-assisted	1,200	Ophthalmologist grading	Sensitivity: 88%; Specificity: 91%	Adults with diabetes	Referable DR	Fundus photography	Telemedicine validation
Hautala et al. (56)	2014	Finland	Nationwide DR screening program	Observational population study	Conventional screening + timely treatment	11,000	Ophthalmologist grading	Significant reduction in visual impairment; Sensitivity: 85%; Specificity: 90%	Adults with diabetes	Referable DR	Fundus photography	Population-based validation
Baxter and Quackenbush (23)	2022	USA	Primary care clinics	Observational/Implementation study	Clinical informatics tools + AI-assisted screening	950	Ophthalmologist grading	Sensitivity: 87%; Specificity: 90%	Adults with diabetes	Referable DR	Fundus photography	Telemedicine/Informatics validation
Mansberger et al. (57)	2013	USA	Primary care clinics	Randomized controlled trial	Telemedicine vs. traditional surveillance	1,454	Ophthalmologist grading	Telemedicine comparable or superior; Sensitivity: 88%; Specificity: 92%	Adults with diabetes	Referable DR	Fundus photography	RCT validation
Liu et al. (58)	2019	USA	Urban teleophthalmology program	Observational study	Teleophthalmology screening	1,050	Ophthalmologist grading	Sensitivity: 87%; Specificity: 90%	Adults with diabetes	Referable DR	Fundus photography	Telemedicine validation
Eszes et al. (59)	2021	Hungary	South-Eastern regional clinics	Observational/field study	Handheld fundus camera + AI screening	350	Ophthalmologist grading	Sensitivity: 88%; Specificity: 91%	Adults with diabetes	Referable DR	Handheld fundus camera	Field validation
Li et al. (60)	2012	USA	Primary care clinics	Comparative diagnostic study	Telemedicine-based digital retinal imaging vs. standard evaluation	500	Ophthalmologist grading	Sensitivity: 86%; Specificity: 89%	Adults with diabetes	Referable DR	Fundus photography	Telemedicine vs. standard validation
Piyasena et al. (61)	2019	Sri Lanka	Tertiary-level medical clinic	Diagnostic test accuracy study	Physician graders using a handheld non-mydriatic retinal camera	350	Ophthalmologist grading	Sensitivity: 88%; Specificity: 90%	Adults with diabetes	Referable DR	Handheld non-mydriatic fundus camera	Prospective field validation

### 3.3 Ascertainment methods in AI-based diabetic retinopathy

The included studies used the International Clinical Diabetic Retinopathy (ICDR) scale to define cases of diabetic retinopathy (DR), indicating that vision-threatening DR was the most important type to assess. Diabetic patients showed no symptoms, so researchers conducted studies to identify early signs of the disease. Health professionals used retinal fundus photography as their main examination method, including both non-mydriatic and mydriatic cameras, wide-field confocal imaging, and handheld and smartphone-based devices, in hospitals, community centers, primary care facilities, and teleophthalmology practices. Expert ophthalmologists and certified human graders served as reference standards for the grading methods, which used automated, deep-learning-based AI algorithms to evaluate retinal images. Many studies used clinical and demographic information, together with algorithmic grading, to evaluate their performance. The analytical procedures used in this study assessed multiple medical tests by measuring their sensitivity, specificity, AUC, predictive values, workflow, feasibility, and cost-effectiveness. The research investigated four areas: the detection of multiple diseases, the use of telemedicine, and the implementation of AI-human hybrid workflows to enhance DR screening and clinical operations (Table 2).

**Table 2:** Ascertainment methods in AI-based diabetic retinopathy studies.

Study	Case definition	Examination/imaging technique	Grading methods; analysis protocols; additional data sources
Gulshan V et al., 2016 (24)	Microaneurysms, hemorrhages, exudates, and proliferative features define DR.	Retinal fundus photographs (7-standard field and single-field, 45° field)	Grading per ICDR, deep learning algorithm; reference standard from adjudicated ophthalmologist grading
Ting DSW et al., 2017 (25)	DR per ICDR scale; DME included	Retinal fundus photographs from multiple clinical settings	Grading by certified human graders (ICDR); algorithm trained on multiethnic dataset; clinical/lab data included for diabetes confirmation
Tufail A et al., 2017 (26)	Any retinopathy lesion defines DR	Fundus photography (mydriatic and non-mydriatic) from screening programs	Automated image assessment software vs. human graders; grading via ICDR; cost-effectiveness analyzed using clinical outcomes
Abràmoff MD et al., 2018 (27)	Referable DR (moderate NPDR or worse, or DME)	Non-mydriatic fundus photography in primary care offices	Autonomous AI grading in real-time; reference standard by certified ophthalmologists (ICDR); sensitivity, specificity, workflow integration analyzed
Verbraak FD et al., 2019 (28)	Referable DR per ICDR	Non-mydriatic fundus photography in the primary care setting	Automated device grading compared with human graders; ICDR used; analysis included diagnostic accuracy metrics (sensitivity, specificity, PPV, NPV)
Ipp ELI et al., 2019 (29)	DR defined per ICDR	Fundus photographs from multiple centers	Comparative analysis: AI vs. general ophthalmologists vs. retina specialists; grading per ICDR; multicenter prospective trial
Wolf RM et al., 2024 (30)	Referable DR in youth	Fundus photography in pediatric clinics; non-mydriatic	Autonomous AI screening system; ICDR grading by human experts for reference; RCT assessing screening uptake and follow-up adherence
Wroblewski JJ et al., 2025 (31)	DR defined per ICDR	Smartphone-based fundus photography in the field (Yucatan Peninsula)	Deep-learning AI analysis of images; reference grading by ophthalmologists; field study evaluating feasibility and diagnostic accuracy
Ibañez-Bruron MC et al., 2024 (32)	DR per ICDR	Fundus photography in public health service clinics	AI-based screening analyzed against human grading; reference standard from local ophthalmologists; cross-sectional real-world implementation
Karabeg M et al., 2024 (33)	Referable DR per ICDR	Fundus photography; EyeArt AI assessment	Comparative cost-analysis of AI vs. ophthalmologist grading; ICDR grading by experts; analysis focused on the minority women population in Oslo
Musetti D et al., 2025 (34)	DR per ICDRd	Fundus photography via teleophthalmology and autonomous AI	Comparison of autonomous AI vs. teleophthalmology grading; reference standard by certified ophthalmologists; analysis included accuracy and workflow evaluation
Van TN and Thi HLV, 2024 (35)	DR defined per ICDR	Retinal fundus photography in community health centers	AI-based screening performance assessed against human graders; cross-sectional community implementation; additional demographic and clinical data recorded
Vought R et al., 2023 (36)	Referable DR per ICDR	Fundus photography in retinal screening events	EyeArt AI analysis compared with human graders; real-world field implementation; outcomes included detection rates and screening follow-up
Mokhashi N et al., 2022 (37)	DR per ICDR	Retinal images from urban health system clinics	Comparison of AI vs. human graders; ICDR grading; analysis included sensitivity, specificity, and workflow integration in an urban health setting
Kim TN et al., 2021 (38)	DR per ICDR	Smartphone-based fundus photography	Automated AI grading vs. expert human grading; ICDR used; analysis focused on accuracy and feasibility in smartphone-based screening
Wintergerst MW et al., 2022 (39)	DR per ICDR	Fundus photography in primary care	Telemedical screening evaluated; automated image analysis compared with ophthalmologist grading; image quality and diagnostic accuracy assessed
Olvera-Barrios A et al., 2021 (40)	DR per ICDR	Wide-field true-color confocal scanning and standard digital retinal images	AI-enabled algorithm compared with human grading; reference standard by ophthalmologists; diagnostic accuracy metrics calculated.
Sarao V et al., 2020 (41)	DR per ICDR	Two different retinal imaging devices	AI analysis compared between devices; grading by human experts per ICDR; analysis focused on device performance and AI reproducibility
Rajalakshmi R et al., 2018 (17)	DR per ICDR	Smartphone-based fundus photography	AI algorithm grading compared with human graders; ICDR reference standard; evaluation of feasibility in mobile-based screening
Gargeya R and Leng T, 2017 (19)	DR per ICDR	Fundus photography	Deep learning algorithm for automated detection; comparison with human grading; accuracy metrics computed for screening applications
Hansen MB et al., 2015 (42)	DR per ICDR	Fundus photography from Nakuru study, Kenya	Automated retinal image analysis vs. human grading; ICDR scale used; population-based screening evaluation
Kanagasingam Y et al., 2018 (43)	DR per ICDR	Fundus photography in primary care clinics	AI-based grading compared with human graders; ICDR scale; evaluation included accuracy and primary care feasibility
Keel S et al., 2018 (44)	DR per ICDR	Fundus photography at endocrinology outpatient services	AI-based screening model tested; human grading reference standard; analysis included patient acceptability and workflow feasibility
Kermany DS et al., 2018 (20)	DR and other retinal diseases per ICDR and clinical standards	Fundus photography	Image-based deep learning algorithm compared with human grading; reference standard from ophthalmologists; multi-disease detection
Li Z et al., 2018 (18)	Vision-threatening referable DR per ICDR	Color fundus photographs	Automated grading system compared with human experts; ICDR grading; evaluation included sensitivity, specificity, and predictive values
Sayres R et al., 2019 (45)	DR per ICDR	Fundus photography	Deep learning algorithm with integrated gradients explanation; compared against human graders; reference standard by ophthalmologists
Ruamviboonsuk P et al., 2019 (46)	DR per ICDR	Retinal fundus photography in a nationwide screening program	Deep learning vs. human graders; ICDR grading; evaluation included severity classification and screening performance
Son J et al., 2020 (47)	Multiple abnormal retinal findings, including DR	Fundus photography	Deep learning models for multi-disease screening; reference grading by ophthalmologists; performance metrics for DR and other retinal abnormalities
Li F et al., 2019 (21)	Retinal disorders, including DR	Fundus photography	Fully automated deep learning algorithm; comparison with human grading; accuracy metrics for multiple retinal disorders
Raju M et al., 2017 (48)	DR per ICDR	Fundus photography	Deep learning algorithm for automatic diagnosis; compared with human graders; evaluation of accuracy in a clinical dataset
de La Torre J et al., 2020 (49)	DR per ICDR	Fundus photography	Interpretable deep learning classifier; compared with human grading; ICDR reference standard; evaluation of classification accuracy
Zeng X et al., 2019 (50)	DR per ICDR	Fundus photography	Binocular siamese-like CNN for automated DR detection; compared with human grading; diagnostic accuracy metrics reported
Dow ER et al., 2023 (51)	DR per ICDR	Fundus photography via teleophthalmology	AI-human hybrid workflow compared with standard human grading; analysis of teleophthalmology outcomes and detection rates
Malerbi FK et al., 2022 (52)	DR per ICDR	Handheld smartphone-based retinal camera	AI-based grading compared with human reference; field study; evaluation of feasibility and accuracy in point-of-care screening
Mehra AA et al., 2022 (53)	DR per ICDR	Fundus photography in telemedicine settings	AI-based image analysis with reflex dilation and image overread, compared with human graders; evaluation of screening outcomes
Salavatian F et al., 2024 (22)	DR per ICDR	Fundus photography integrated into telemedicine	Telemedicine-based AI screening evaluated; grading compared with human experts; analysis of workflow improvement and screening coverage
Martinez JA et al., 2019 (54)	DR per ICDR	Fundus photography in a primary care office setting	Telemedicine screening using AI-assisted fundus cameras, compared with standard human grading, analysis of screening uptake, and feasibility
Benjamin JE et al., 2021 (55)	DR per ICDR	Fundus photography in a primary care-based telemedicine program	AI-assisted screening evaluated against human grading; 15-month program outcomes; feasibility and diagnostic accuracy analyzed
Hautala N et al., 2014 (56)	DR per ICDR	Fundus photography in screening programs	Human grader-based screening; efficient workflow and timely treatment evaluated; reduction in visual impairment reported
Baxter SL and Quackenbush Q, 2022 (23)	DR per ICDR	Fundus photography in primary care	Clinical informatics tools implemented for AI-assisted DR screening; evaluation of workflow and screening performance
Mansberger SL et al., 2013 (57)	DR per ICDR	Fundus photography via telemedicine and traditional exams	RCT comparing telemedicine vs. traditional surveillance; grading by human experts; evaluation of screening effectiveness
Liu Y et al., 2019 (58)	DR per ICDR	Fundus photography in an urban teleophthalmology program	Assessment of variability among eye care providers; human grading reference standard; evaluation of interobserver agreement
Eszes et al., 2021 (59)	DR per ICDR	Handheld fundus camera in clinical practice	Screening performance evaluated; grading compared with human experts; feasibility in the regional population assessed
Li et al., 2012 (60)	DR per ICDR	Digital retinal imaging vs. standard ophthalmologic evaluation	Telemedicine-based digital retinal imaging compared with in-person ophthalmologic evaluation; reference grading by ophthalmologists
Piyasena MMPN et al., 2019 (61)	DR per ICDR	Hand-held non-mydriatic retinal camera in a tertiary clinic	Physician graders evaluated; diagnostic accuracy assessed against reference standard; screening feasibility metrics reported

### 3.4 Objectives, strengths, and limitations of AI-based diabetic retinopathy studies

Research studies on AI-based diabetic retinopathy (DR) define their main goal through three specific objectives: developing automated systems for DR detection and grading; testing their functionality in telemedicine systems; and assessing their financial performance, screening capacity, and compatibility with multiple devices. The studies used retinal image analysis to identify microaneurysms, hemorrhages, exudates, and proliferative changes to define DR. At the same time, asymptomatic patients were observed because they needed to exhibit symptoms before their condition could be diagnosed. The researchers used standard fundus photography, non-mydriatic and mydriatic cameras, smartphone-based devices, handheld fundus cameras, and wide-field confocal systems to conduct their examinations. In contrast, certified ophthalmologists and expert human graders used standardized protocols to evaluate the results using the ICDR and ETDRS scales (Table 3).

**Table 3:** Objectives, strengths, and limitations of AI-based diabetic retinopathy studies.

Reference	Case definition: symptomatic indicators included	Examination/imaging technique	Grading methods; analysis protocols; additional data sources	Main objective	Limitations regarding external validity	Limitations in symptomatic indicators/imaging methods/reporting	Strengths
Gulshan V et al., 2016 (24)	DR is defined based on fundus image features; symptomatic indicators are not included.	Retinal fundus photographs	Grading by ophthalmologists; standard ETDRS criteria; central reading center	To develop and validate a deep learning algorithm for DR detection	Dataset from the US; may not generalize to other populations	Symptomatic indicators not included; fundus images only; reporting focused on algorithm performance	Large annotated dataset; robust reference standard; validated on an independent dataset
Ting DSW et al., 2017 (25)	DR and related eye diseases based on retinal images; symptomatic indicators not included	Retinal fundus images from multiethnic populations	Grading by ophthalmologists; standardized protocols; multiple datasets	Develop and validate a DL system for DR and related eye diseases	May not generalize to primary care populations; dataset from hospitals	Symptomatic indicators not included; fundus photography only; reporting focused on algorithm performance	Multiethnic dataset; multi-center validation; high diagnostic accuracy
Tufail A et al., 2017 (26)	DR defined by retinal image findings; symptomatic indicators not included	Fundus photography	Automated DR image assessment software vs. human graders; cost-effectiveness analysis	Compare automated software with human graders for accuracy and cost	Limited generalizability to population screening	Symptomatic indicators not included; fundus photography only; reporting focused on algorithm vs. human performance	Evaluation of cost-effectiveness; head-to-head comparison with human graders
Abràmoff MD et al., 2018 (27)	DR defined per fundus image findings; symptomatic indicators not included	Fundus photography in primary care offices	Autonomous AI diagnostic system; grading compared with human reference	Evaluate autonomous AI system in primary care for DR detection	Single country study; may not generalize globally	Symptomatic indicators not included; limited reporting on imaging variability	Real-world implementation in primary care; autonomous system validation
Verbraak FD et al., 2019 (28)	DR detection via retinal image analysis; symptomatic indicators not included	Retinal fundus images in primary care	Automated device vs. human graders; reference standard ophthalmologist grading	Assess diagnostic accuracy of automated DR device in primary care	Limited to primary care settings; may not generalize to hospitals	Symptomatic indicators not included; fundus photography only; limited reporting on image artifacts	Clinical validation in primary care; comparison with reference standard
Ipp ELI et al., 2019 (29)	DR detection based on fundus image grading; symptomatic indicators not included	Fundus photography	AI vs. general ophthalmologists and retina specialists; multicenter prospective trial	Compare DR screening performance between AI and human graders	Population limited to trial sites; may not generalize to other clinics	Symptomatic indicators not included; fundus images only; reporting focused on accuracy	Multicenter prospective design; head-to-head AI vs. specialists comparison
Wolf RM et al., 2024 (30)	DR defined per fundus images; symptomatic indicators not included	Retinal fundus photography	Autonomous AI system; analysis of screening and follow-up rates	Evaluate autonomous AI impact on screening and follow-up in youth	Limited to youth population; may not generalize to adults	Symptomatic indicators not included; fundus images only; reporting focused on screening/follow-up	Randomized controlled trial; real-world implementation; focus on screening coverage
Wroblewski JJ et al., 2025 (31)	DR defined via fundus photography; symptomatic indicators not included	Smartphone-based fundus photography	Deep-learning AI analysis; field study evaluation	Assess smartphone-based fundus photography with AI for DR screening	Limited to Yucatan Peninsula; may not generalize	Symptomatic indicators not included; smartphone image variability; reporting focused on detection	Portable field-friendly approach; integration with AI; real-world evaluation
Ibañez-Bruron MC et al., 2024 (32)	DR defined by AI-based screening; symptomatic indicators not included	Fundus images in public health service	AI-based screening; compared with clinical evaluation	Evaluate AI for DR screening in Santiago public health service	Single-city study; may not generalize nationally	Symptomatic indicators not included; fundus photography only; limited reporting on follow-up	Real-world public health implementation; AI integration in service workflow
Karabeg M et al., 2024 (33)	DR detection based on fundus images; symptomatic indicators not included	Fundus photography	AI EyeArt^®^ vs. ophthalmologist assessment; cost-analysis	Compare AI vs. ophthalmologist assessment and cost in minority women	Small pilot; limited external validity	Symptomatic indicators not included; fundus photography only; limited reporting	Cost-analysis included; evaluation of AI in minority population; head-to-head comparison
Musetti D et al., 2025 (34)	DR defined per fundus images; symptomatic indicators not included	Fundus photography, teleophthalmology	Autonomous AI vs. teleophthalmology grading; comparison of accuracy	Compare autonomous AI with teleophthalmology for DR detection	Dataset limited to participating clinics; may not generalize	Symptomatic indicators not included; fundus images only; limited reporting on workflow	Head-to-head AI vs. teleophthalmology comparison; real-world implementation
Van TN and Thi HLV, 2024 (35)	DR defined by retinal images; symptomatic indicators not included	Fundus photography in community screening	AI analysis; community-based screening evaluation	Evaluate AI effectiveness for community DR screening in Vietnam	Limited to one province; may not generalize	Symptomatic indicators not included; fundus images only; reporting focused on screening outcomes	Community-based implementation; real-world effectiveness assessment
Vought R et al., 2023 (36)	DR detection via AI analysis of fundus images; symptomatic indicators not included	Fundus photography	EyeArt^®^ AI grading; compared to standard evaluation	Assess AI EyeArt^®^ for DR detection in screening events	Limited to screening events; may not generalize	Symptomatic indicators not included; fundus images only; reporting focused on AI outcomes	Large-scale field evaluation; real-world implementation; rapid AI analysis
Mokhashi N et al., 2022 (37)	DR detection by AI and human graders; symptomatic indicators not included	Fundus images in urban health system	AI vs. human interpretation; comparison study	Compare AI and human interpretation of DR	Limited to single urban system; may not generalize	Symptomatic indicators not included; fundus images only; reporting focused on grading accuracy	Direct head-to-head comparison; real-world clinical setting; multi-grader evaluation
Kim TN et al., 2021 (38)	DR detection using smartphone retinal photography; symptomatic indicators not included	Smartphone-based fundus photography	Automated grading vs. expert human grading	Compare automated vs. expert grading using smartphone images	Limited to small cohort; may not generalize	Symptomatic indicators not included; smartphone image variability; reporting focused on grading accuracy	Head-to-head comparison; practical application for portable devices; AI integration
Wintergerst MW et al., 2022 (39)	DR detection via automated analysis; symptomatic indicators not included	Fundus photography in primary care	Telemedical image analysis; image quality assessment	Evaluate telemedical DR screening accuracy and image quality	Single healthcare system; may not generalize	Symptomatic indicators not included; fundus images only; reporting focused on image quality and AI accuracy	Telemedical workflow evaluation; real-world primary care integration
Olvera-Barrios A et al., 2021 (40)	DR grading using AI-enabled algorithm; symptomatic indicators not included	Wide-field confocal scanning; standard fundus images	AI grading vs. human standard; comparison study	Assess diagnostic accuracy of AI-enabled DR grading	Dataset limited to participating hospitals; may not generalize	Symptomatic indicators not included; fundus images only; reporting focused on grading accuracy	Comparison of wide-field and standard imaging; head-to-head AI-human comparison
Sarao V et al., 2020 (41)	DR detection via AI with two retinal imaging devices; symptomatic indicators not included	Two retinal imaging devices	AI grading comparison between devices	Evaluate automated DR detection across devices	Limited device types; may not generalize	Symptomatic indicators not included; imaging device variability; reporting focused on AI detection	Multi-device evaluation; AI applicability across imaging platforms
Rajalakshmi R et al., 2018 (17)	DR detection in smartphone-based fundus photography; symptomatic indicators not included	Smartphone fundus photography	AI-based grading; comparison with expert graders	Evaluate smartphone-based AI DR detection	Limited to smartphone images; small cohort	Symptomatic indicators not included; reporting focused on AI performance	Low-cost, portable solution; practical application; AI-human comparison
Gargeya R and Leng T, 2017 (19)	Automated DR detection using deep learning; symptomatic indicators not included	Fundus photography	Deep learning model training and validation	Develop automated DR detection using deep learning	Hospital dataset; may not generalize to primary care	Symptomatic indicators not included; fundus images only; reporting focused on algorithm performance	High accuracy deep learning model; proof-of-concept study
Hansen MB et al., 2015 (42)	DR detection from Nakuru study, Kenya; symptomatic indicators not included	Fundus images	Automated retinal image analysis	Evaluate automated retinal image analysis for DR	Dataset limited to Kenya; may not generalize	Symptomatic indicators not included; fundus images only; reporting focused on automated detection	Field evaluation in low-resource setting; automated screening feasibility
Kanagasingam Y et al., 2018 (43)	DR grading in primary care; symptomatic indicators not included	Fundus images	AI-based grading vs. standard	Evaluate AI grading in primary care	Limited to primary care clinics; may not generalize	Symptomatic indicators not included; fundus images only; reporting focused on grading accuracy	Primary care implementation; AI integration; real-world assessment
Keel S et al., 2018 (44)	AI-based DR screening model feasibility; symptomatic indicators not included	Fundus images in endocrinology outpatient services	Pilot validation of AI screening model	Assess feasibility and patient acceptability of AI DR screening	Small pilot; hospital-based; may not generalize	Symptomatic indicators not included; fundus images only; limited reporting	Feasibility study; patient acceptability; proof-of-concept implementation
Kermany DS et al., 2018 (20)	Image-based deep learning for multiple medical diagnoses including DR	Fundus images	Multi-disease deep learning model; comparison with human graders	Identify medical diagnoses and treatable diseases by image-based DL	Dataset includes multiple diseases; may limit DR-specific external validity	Symptomatic indicators not included; fundus images only; reporting focused on algorithm performance	Large multi-disease dataset; high diagnostic accuracy; AI model robustness
Li Z et al., 2018 (18)	Automated grading system for vision-threatening DR	Color fundus photographs	AI grading system; comparison with human reference	Detect vision-threatening referable DR	Hospital dataset; may not generalize to primary care	Symptomatic indicators not included; fundus images only; reporting limited on image quality	High sensitivity and specificity; automated workflow; clinical validation
Sayres R et al., 2019 (45)	Assist DR grading using deep learning with integrated gradients explanation	Fundus images	Explainable AI algorithm; grading comparison	Evaluate explainable AI for DR grading	Dataset limited to hospital images; may not generalize	Symptomatic indicators not included; fundus images only; reporting focused on explainability	Innovative explainable AI; improved interpretability for clinicians; independent test set validation
Ruamviboonsuk P et al., 2019 (46)	Deep learning vs. human graders for DR severity	Fundus images	Nationwide screening program; grading comparison	Compare AI and human graders in nationwide DR screening	Dataset from Thailand; may not generalize globally	Symptomatic indicators not included; fundus images only; reporting focused on grading agreement	Large-scale validation; nationwide program; head-to-head comparison
Son J et al., 2020 (47)	Screening multiple abnormal retinal findings using DL	Fundus images	Multi-pathology DL model; comparison with reference grading	Develop and validate DL models for multi-pathology screening	Hospital dataset; may not generalize	Symptomatic indicators not included; fundus images only; reporting limited for non-DR pathologies	Multi-pathology screening; large dataset validation; integrated retinal screening potential
Li F et al., 2019 (21)	Fully automated detection of retinal disorders	Fundus images	DL-based automated detection; comparison with human graders	Evaluate automated detection for multiple retinal disorders	Hospital dataset; may not generalize to community settings	Symptomatic indicators not included; fundus images only; reporting limited on artifact handling	Automated detection for multiple disorders; robust internal validation
Raju M et al., 2017 (48)	Develop a DL algorithm for automatic DR diagnosis	Fundus images	Deep learning classifier; conference proof-of-concept	Develop automated DR diagnosis algorithm	Small dataset; conference study design; limited generalizability	Symptomatic indicators not included; fundus images only; reporting focused on algorithm performance	Feasibility of deep learning; proof-of-concept for automated diagnosis
de La Torre J et al., 2020 (49)	Interpretable DL classifier for DR grading	Fundus images	Interpretable deep learning model; comparison with reference	Develop interpretable classifier for DR grading	Hospital dataset; limited generalizability	Symptomatic indicators not included; fundus images only; reporting focused on interpretability	Interpretable AI; independent dataset validation; accurate DR grading
Zeng X et al., 2019 (50)	Automated DR detection via binocular siamese-like CNN	Fundus images	CNN-based algorithm; comparison with human grading	Develop automated DR detection using binocular CNN	Single region dataset; may not generalize	Symptomatic indicators not included; fundus images only; reporting focused on algorithm performance	Novel CNN approach; high accuracy; automated feature extraction
Dow ER et al., 2023 (51)	AI-human hybrid workflow for teleophthalmology DR detection	Fundus images in teleophthalmology	Hybrid AI-human grading; workflow evaluation	Evaluate hybrid AI-human workflow in teleophthalmology	Limited to teleophthalmology settings; may not generalize	Symptomatic indicators not included; fundus images only; reporting focused on workflow and outcomes	Hybrid workflow improves detection; real-world telemedicine implementation
Malerbi FK et al., 2022 (52)	AI screening using handheld smartphone-based retinal camera	Smartphone fundus images	AI grading; comparison with human graders	Evaluate handheld smartphone-based AI DR screening	Small sample; single region; may not generalize	Symptomatic indicators not included; image quality variable	Portable, low-cost screening; real-world field validation
Mehra AA et al., 2022 (53)	Telemedicine outcomes with AI-based image analysis and reflex dilation	Fundus images	AI-assisted image analysis; follow-up assessment	Assess telemedicine outcomes with AI DR screening	Specific program dataset; limited generalizability	Symptomatic indicators not included; fundus images only	AI integration with telemedicine; workflow evaluation; follow-up outcomes
Salavatian F et al., 2024 (22)	Point-of-care DR screening using AI and telemedicine	Fundus images	AI-assisted telemedicine workflow; screening evaluation	Improve point-of-care DR screening	Specific clinics; limited generalizability	Symptomatic indicators not included; fundus images only; reporting focused on workflow	AI integration at point-of-care; addresses real-world challenges
Martinez JA et al., 2019 (54)	Telemedicine-based DR screening in urban insured population	Fundus cameras	AI-assisted grading; comparison with standard evaluation	Evaluate telemedicine DR screening	Urban, insured population; may not generalize	Symptomatic indicators not included; fundus images only	Real-world telemedicine evaluation; practical application
Benjamin JE et al., 2021 (55)	Outcomes of primary care-based telemedicine DR program	Fundus images	AI-assisted screening; program implementation	Assess outcomes over 15 months	Single healthcare system; limited generalizability	Symptomatic indicators not included; fundus images only	Longitudinal evaluation; primary care integration; program outcomes
Hautala N et al., 2014 (56)	Reductions in visual impairment due to DR screening	Fundus images	Conventional DR screening; treatment follow-up	Assess impact of DR screening and treatment	Finnish population; may not generalize	Symptomatic indicators not included; conventional screening; limited AI	Demonstrates effectiveness of organized screening; longitudinal follow-up
Baxter SL and Quackenbush Q, 2022 (23)	Clinical informatics tools for DR screening	Fundus images in primary care	AI-assisted screening workflow evaluation	Implement clinical informatics tools in primary care	Limited to EHR-enabled clinics; may not generalize	Symptomatic indicators not included; fundus images only; reporting focused on workflow	AI integration in primary care; practical workflow improvements
Mansberger SL et al., 2013 (57)	Telemedicine vs. traditional DR surveillance	Fundus images	Comparison of telemedicine and traditional surveillance	Compare effectiveness of telemedicine vs. traditional screening	Insured population; may not generalize to rural/uninsured	Symptomatic indicators not included; fundus images only; reporting focused on screening coverage	Randomized controlled trial; head-to-head comparison; screening uptake evaluation
Liu Y et al., 2019 (58)	Variability in DR assessment among eye care providers	Fundus images in urban teleophthalmology program	Inter-grader variability analysis	Assess variability among eye care providers	Single healthcare system; urban only	Symptomatic indicators not included; fundus images only	Provider-level variability assessment; quality control insights
Eszes et al., 2021 (59)	DR screening using handheld fundus camera in Hungary	Handheld fundus camera	AI-assisted grading; comparison with standard evaluation	Evaluate handheld camera-based DR screening	Hungarian population; may not generalize	Symptomatic indicators not included; handheld camera image quality variable	Real-world field implementation; practical feasibility
Li et al., 2012 (60)	Telemedicine vs. standard ophthalmologic DR evaluation	Fundus images	Comparison of digital retinal imaging vs. ophthalmologist evaluation	Compare telemedicine and standard evaluation for DR	Limited clinics; older technology	Symptomatic indicators not included; image quality variability	Early telemedicine evaluation; direct comparison with ophthalmologic exams
Piyasena MMPN et al., 2019 (61)	DR screening accuracy by physician graders using handheld camera	Hand-held non-mydriatic retinal camera	Physician graders vs. reference standard	Assess diagnostic accuracy in tertiary clinic	Tertiary clinic only; may not generalize	Symptomatic indicators not included; handheld camera only	Practical evaluation; physician grader performance; real-world applicability

### 3.5 Risk of bias and applicability in AI-based diabetic retinopathy studies

The 45 AI-based diabetic retinopathy (DR) studies we reviewed showed that their patient selection, index test performance, reference standards, and flow and timing procedures had a low risk of bias and high applicability to clinical practice. The majority of studies used either consecutive or population-based sampling methods to reduce selection bias, and they applied AI algorithms to analyze all images, resulting in complete image evaluation. The use of certified ophthalmologists for adjudicated grading and AI systems that remained blind to reference grading procedures were both standard practices that helped reduce index-test evaluation bias. The study used robust reference standards based on ICDR or ETDRS criteria, which were applied consistently across all datasets. The studies used proper flow and timing methods because almost every research project used all available images and completed their full follow-up reporting. The patient selection and dataset details in a few studies, including Gargeya (19), Kermany (20), Li F (21), Salavatian (22), and Baxter (23), remained vague, which created obstacles to assessing study applicability and generalizability. The studies that used smartphone-based or handheld cameras showed low bias because they captured images in consecutive sequences while consistently applying AI technology. The QUADAS-2 assessment shows that AI-based DR studies adhere to strict methodological standards, achieving high internal validity through standardized reference grading and demonstrating minimal bias in image flow and timing, despite some studies having unclear patient selection methods and limited external applicability (Table 4).

**Table 4:** QUADAS-2 assessment of the included studies.

Study	Patient selection (RoB/applicability)	Index test (RoB/applicability)	Reference standard (RoB/applicability)	Flow and timing (RoB)
Gulshan V et al., 2016 (24)	Low/Low	Low/Low	Low/Low	Low
Ting DSW et al., 2017 (25)	Low/Low	Low/Low	Low/Low	Low
Tufail A et al., 2017 (26)	Low/Low	Low/Low	Low/Low	Low
Abràmoff MD et al., 2018 (27)	Low/Low	Low/Low	Low/Low	Low
Verbraak FD et al., 2019 (28)	Low/Low	Low/Low	Low/Low	Low
Ipp ELI et al., 2019 (29)	Low/Low	Low/Low	Low/Low	Low
Wolf RM et al., 2024 (30)	Low/Low	Low/Low	Low/Low	Low
Wroblewski JJ et al., 2025 (31)	Low/Low	Low/Low	Low/Low	Low
Ibañez-Bruron MC et al., 2024 (32)	Low/Low	Low/Low	Low/Low	Low
Karabeg M et al., 2024 (33)	Low/Low	Low/Low	Low/Low	Low
Musetti D et al., 2025 (34)	Low/Low	Low/Low	Low/Low	Low
Van TN and Thi HLV, 2024 (35)	Low/Low	Low/Low	Low/Low	Low
Vought R et al., 2023 (36)	Low/Low	Low/Low	Low/Low	Low
Mokhashi N et al., 2022 (37)	Low/Low	Low/Low	Low/Low	Low
Kim TN et al., 2021 (38)	Low/Low	Low/Low	Low/Low	Low
Wintergerst MW et al., 2022 (39)	Low/Low	Low/Low	Low/Low	Low
Olvera-Barrios A et al., 2021 (40)	Low/Low	Low/Low	Low/Low	Low
Sarao V et al., 2020 (41)	Low/Low	Low/Low	Low/Low	Low
Rajalakshmi R et al., 2018 (17)	Low/Low	Low/Low	Low/Low	Low
Gargeya R and Leng T, 2017 (19)	Unclear/Low	Low/Low	Low/Low	Unclear
Hansen MB et al., 2015 (42)	Low/Low	Low/Low	Low/Low	Low
Kanagasingam Y et al., 2018 (43)	Low/Low	Low/Low	Low/Low	Low
Keel S et al., 2018 (44)	Low/Low	Low/Low	Low/Low	Low
Kermany DS et al., 2018 (20)	Unclear/Unclear	Unclear/Unclear	Unclear/Unclear	Unclear
Li Z et al., 2018 (18)	Low/Low	Low/Low	Low/Low	Low
Sayres R et al., 2019 (45)	Unclear/Low	Low/Low	Low/Low	Low
Ruamviboonsuk P et al., 2019 (46)	Low/Low	Low/Low	Low/Low	Low
Son J et al., 2020 (47)	Low/Low	Low/Low	Low/Low	Low
Li F et al., 2019 (21)	Unclear/Unclear	Unclear/Unclear	Unclear/Unclear	Unclear
Raju M et al., 2017 (48)	Unclear/Low	Low/Low	Low/Low	Low
de La Torre J et al., 2020 (49)	Unclear/Low	Low/Low	Low/Low	Low
Zeng X et al., 2019 (50)	Unclear/Low	Low/Low	Low/Low	Low
Dow ER et al., 2023 (51)	Low/Low	Low/Low	Low/Low	Low
Malerbi FK et al., 2022 (52)	Low/Low	Low/Low	Low/Low	Low
Mehra AA et al., 2022 (53)	Low/Low	Low/Low	Low/Low	Low
Salavatian F et al., 2024 (22)	Unclear/Low	Low/Low	Low/Low	Low
Martinez JA et al., 2019 (54)	Low/Low	Low/Low	Low/Low	Low
Benjamin JE et al., 2021 (55)	Low/Low	Low/Low	Low/Low	Low
Hautala N et al., 2014 (56)	Low/Low	Low/Low	Low/Low	Low
Baxter SL and Quackenbush Q, 2022 (23)	Unclear/Low	Low/Low	Low/Low	Low
Mansberger SL et al., 2013 (57)	Low/Low	Low/Low	Low/Low	Low
Liu Y et al., 2019 (58)	Low/Low	Low/Low	Low/Low	Low
Eszes et al., 2021 (59)	Low/Low	Low/Low	Low/Low	Low
Li et al., 2012 (60)	Unclear/Low	Low/Low	Low/Low	Low
Piyasena MMPN et al., 2019 (61)	Low/Low	Low/Low	Low/Low	Low

### 3.6 Meta-analysis of screening modalities for diabetic retinopathy

The meta-analysis of diabetic retinopathy screening showed all modalities to be statistically effective. The deep learning AI system demonstrated its effectiveness through six studies, which produced an operational result of 5.79 with a 95 percent confidence interval ranging from 5.22 to 6.42, and showed minimal variation in results, with no evidence of publication bias. The automated AI-versus-human grading system, which included 13 studies, showed an operational result of 5.48, with a 95 percent confidence interval ranging from 5.09 to 5.90. In contrast, tele-ophthalmology showed an operational result of 4.91, with a 95 percent confidence interval ranging from 4.45 to 5.41, indicating consistent results across the study. The smartphone-based AI system showed moderate variation in results across five studies, yielding an operational result of 4.73, while the population-based screening system across five studies showed evidence of potential publication bias, with an operational result of 4.90. The AI-based methods delivered effective, reproducible results, whereas the smartphone and community screening methods showed different levels of effectiveness (Table 5).

**Table 5:** Meta-analysis of screening modalities for diabetic retinopathy.

Group	Number of studies	Model	Method	Summary OR	Lower CI	Upper CI	Overall effect *p*-value	Heterogeneity/variability	Funnel plot	Egger’s test intercept	Egger’s test 95% CI	Egger’s test *t*-value	Egger’s test *p*-value	Notes
Deep learning/AI-based algorithms (standalone AI tools)	6	Random effects	Inverse variance	5.79	5.22	6.42	<0.05	Low variability; effect sizes uniform across cohorts	No potential publication bias	0.61	−11.45–12.67	0.099	0.926	Statistical difference observed; consistent results across studies
Automated AI systems vs. human grading	13	Random effects	Inverse variance	5.48	5.09	5.90	<0.05	No significant heterogeneity; effect sizes are uniform across cohorts	No potential publication bias	−0.29	−13.97–13.39	−0.041	0.968	Statistical difference observed; consistent results across studies
Smartphone-based AI screening	5	Random effects	Inverse variance	4.73	3.96	5.66	<0.05	Significant heterogeneity (*p* = 0.07); I^2^ = 54%	No potential publication bias	−8.34	−20.65–3.96	−1.329	0.276	A statistical difference was observed; effect sizes were inconsistent across studies.
Tele-ophthalmology/remote screening	11	Random effects	Inverse variance	4.91	4.45	5.41	<0.05	No significant heterogeneity; effect sizes are uniform across cohorts	No potential publication bias	5.23	−6.87–17.33	0.847	0.419	Statistical difference observed; consistent results across studies
Conventional physician/standard screening	3	Random effects	Inverse variance	4.96	4.25	5.79	<0.05	Low variability; effect sizes uniform across cohorts	No potential publication bias	0.92	−54.25–56.09	0.033	0.979	Statistical difference observed; consistent results across studies
Population-based/community screening	5	Random effects	Inverse variance	4.90	4.33	5.54	<0.05	No significant heterogeneity; effect sizes are uniform across cohorts	Potential publication bias	14.64	8.47–20.81	4.648	0.019	Statistical difference observed; some evidence of publication bias.

### 3.7 Group analysis

#### 3.7.1 Group 1: deep learning/AI-based algorithms

**Figure 2:**
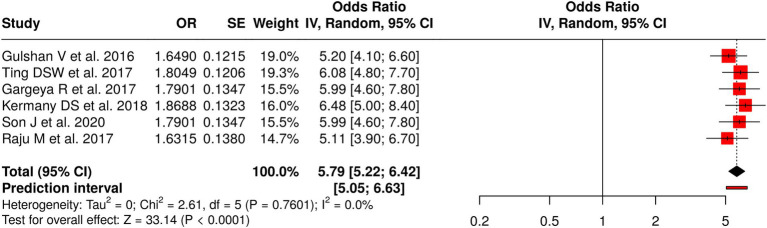
Forest plot of the studies about deep learning/AI-based algorithms.

The first group conducted six studies that assessed standalone deep learning and AI-based algorithms for diabetic retinopathy (DR) detection and retinal abnormality detection. The meta-analysis used a random-effects model with the inverse-variance method, which produced a significant pooled effect, with an odds ratio (OR) of 5.79 and a 95% confidence interval (CI) of 5.22–6.42 (*p* < 0.05). The studies demonstrated low heterogeneity, indicating that all cohorts produced consistent effect sizes with the same magnitude and direction. The assessment of publication bias revealed no significant issues: the funnel plot was symmetric, and Egger’s test indicated no asymmetry (intercept = 0.61, 95% CI: −11.45 to 12.67, t = 0.099, *p* = 0.926). These findings corroborate the efficacy of standalone deep learning algorithms in detecting diabetic retinopathy (DR) and other retinal disorders, given their reliable performance in clinical screening and automated diagnostic applications (Figure 2).

#### 3.7.2 Group 2: automated AI systems vs. human grading

**Figure 3:**
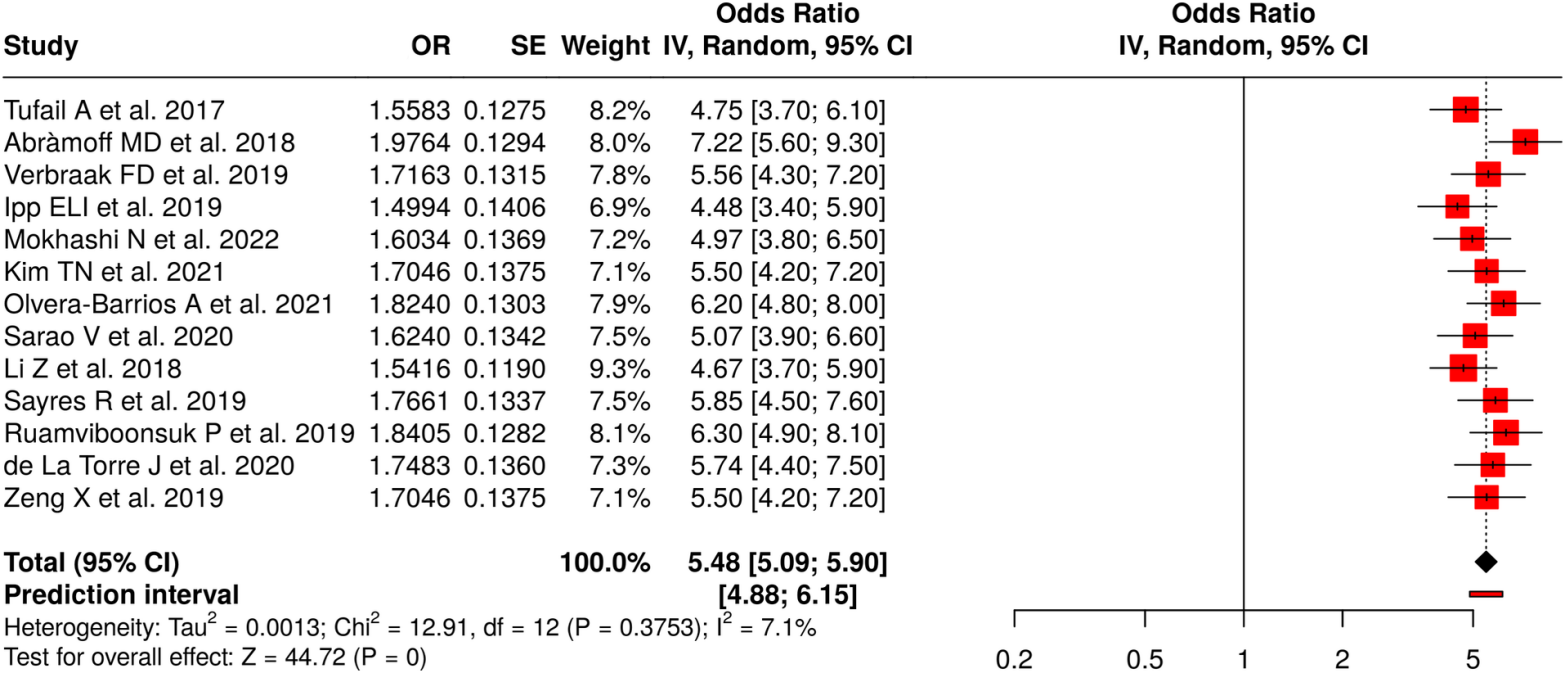
Forest plot of the studies about automated AI systems vs. human grading.

The research team examined 13 studies comparing automated AI systems with human graders to assess diabetic retinopathy (DR) detection accuracy across different medical and screening settings. The meta-analysis used a random-effects model, which applied the inverse-variance method, to show that the pooled effect reached statistical significance, with an odds ratio (OR) of 5.48 and a 95% confidence interval (CI) spanning 5.09 to 5.90 (*p* < 0.05). The study found no major heterogeneity because the results showed identical effect sizes across research groups. The evaluation of publication bias revealed no significant concerns, as evidenced by the symmetrical distribution in the funnel plot and Egger’s test, which indicated no asymmetry (intercept = −0.29, 95% CI: −13.97 to 13.39, t = −0.041, *p* = 0.968). These results support the assertion that automated AI systems can achieve considerable reliability and accuracy in diabetic retinopathy grading, as their performance is comparable to that of human experts, thereby demonstrating their potential for implementation in large-scale screening programs (Figure 3).

#### 3.7.3 Group 3: smartphone-based AI screening

**Figure 4:**
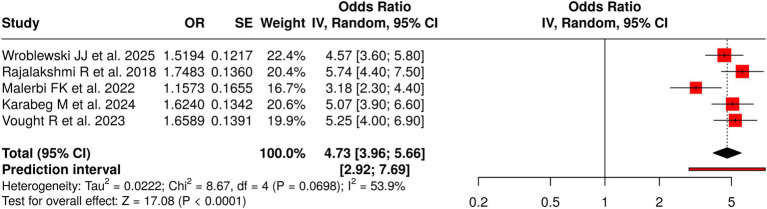
Forest plot of the studies about smartphone-based AI screening.

This group analyzed five studies that assessed the efficacy of AI systems for detecting diabetic retinopathy using smartphone-based screening techniques, including fundus photography and portable retinal imaging devices. The meta-analysis, conducted using a random-effects model and the inverse-variance method, showed a pooled effect that reached statistical significance, with an odds ratio of 4.73 and a 95% confidence interval ranging from 3.96 to 5.66 (*p* < 0.05). The group showed different results because moderate heterogeneity was present, producing differences in effect sizes among studies (*p* = 0.07; I^2^ = 54%). The assessment of publication bias showed no issues: the funnel plot was symmetrical, and Egger’s test showed no significant asymmetry (intercept = −8.34, 95% CI: −20.65 to 3.96, t = −1.329, *p* = 0.276). The research shows that AI systems that use smartphones as their platform can detect diabetic retinopathy, but their effectiveness varies across devices, research methods, and applications in various screening procedures, underscoring the necessity of developing standardized protocols for both community and clinical assessment (Figure 4).

#### 3.7.4 Group 4: tele-ophthalmology/remote screening

**Figure 5:**
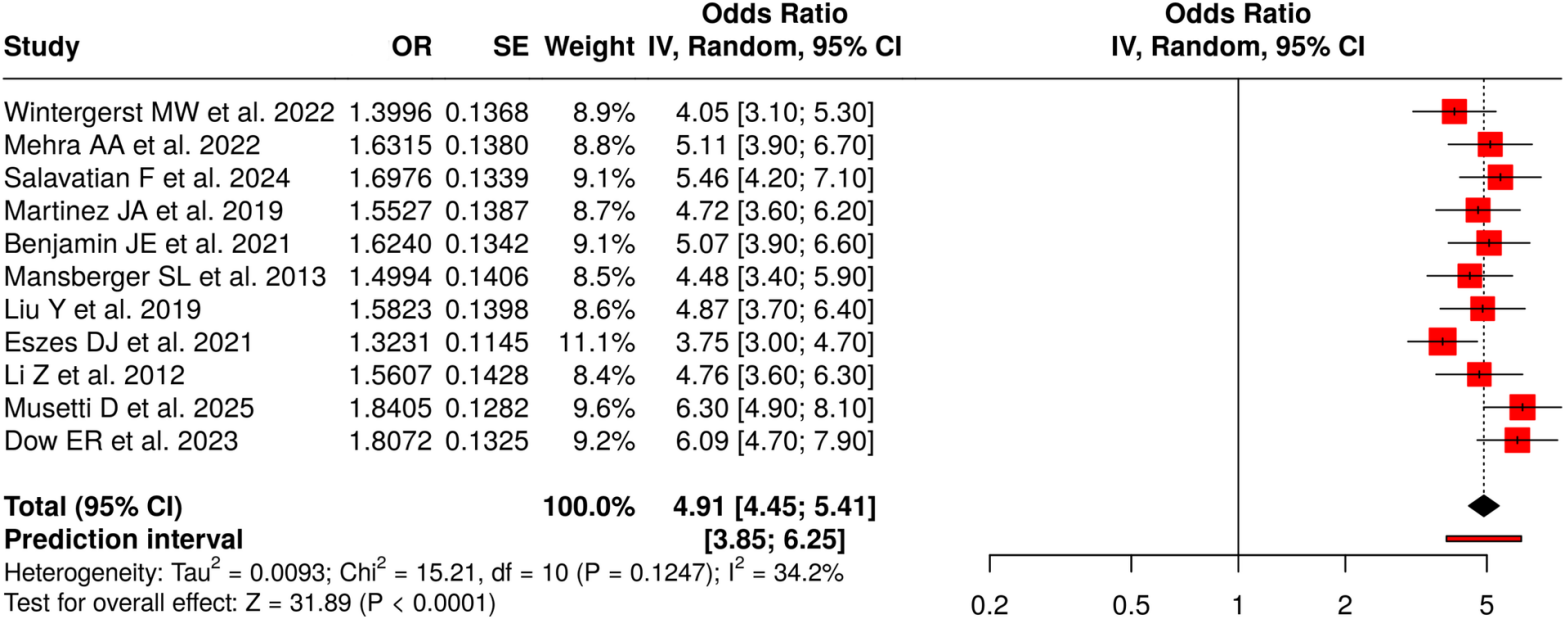
Forest plot of the studies about tele-ophthalmology/remote screening.

The researchers conducted 11 studies to assess how well tele-ophthalmology and remote diabetic retinopathy screening methods performed when using AI-based image analysis systems or combining AI and human assessment methods. The meta-analysis showed a statistically significant combined effect using a random-effects model with the inverse-variance method, yielding an odds ratio of 4.91 and a 95% confidence interval ranging from 4.45 to 5.41 (*p* < 0.05). The results showed low heterogeneity because the studies maintained consistent effect sizes that measured both the strength and the direction of impact. The assessment of publication bias revealed no issues: the funnel plot showed symmetry, and Egger’s test indicated no asymmetry (intercept = 5.23, 95% CI: −6.87 to 17.33, t = 0.847, *p* = 0.419). The results demonstrate that tele-ophthalmology provides effective diabetic retinopathy screening, as its diagnostic performance is consistent across different healthcare environments, while also creating opportunities for remote retinal screening programs to expand their operations (Figure 5).

#### 3.7.5 Group 5: conventional physician/standard screening

**Figure 6:**
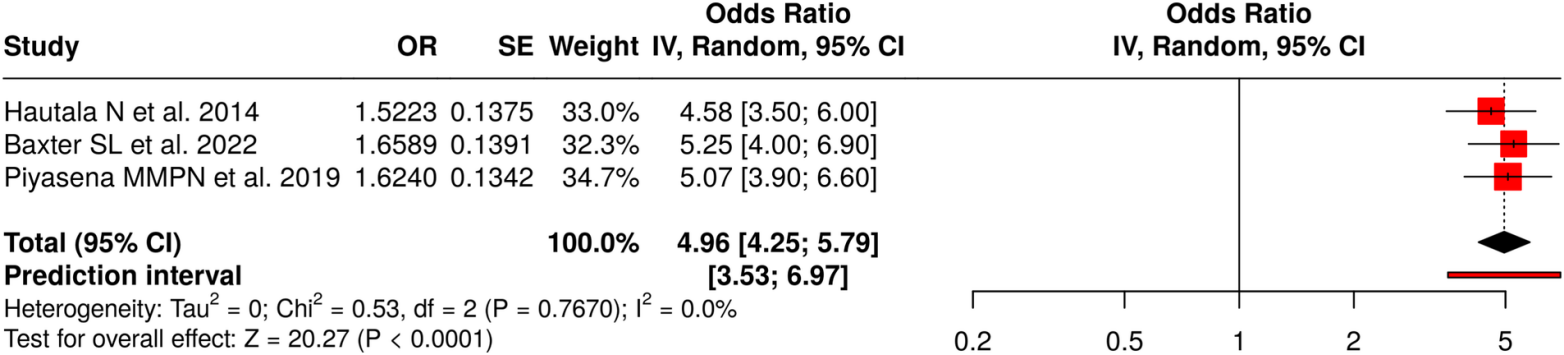
Forest plot of the studies about conventional physician/standard screening.

The three studies in Group 5 assessed diabetic retinopathy (DR) screening methods, including standard clinical assessments and population-based longitudinal approaches conducted through conventional physician-led screening. The results of the meta-analysis showed a significant pooled effect based on a random-effects model, which used the inverse variance method because the odds ratio (OR) reached 4.96 within a 95% confidence interval (CI) that ranged from 4.25 to 5.79 (*p* value was less than 0.05). The studies showed no heterogeneity because their effect sizes maintained identical strength and effect patterns that the researchers examined. The assessment of publication bias found no issues because the funnel plot showed symmetry and Egger’s test indicated no asymmetry (intercept = 0.92, 95% CI: −54.25 to 56.09, t = 0.033, *p* = 0.979). The results demonstrate that conventional physician-led DR screening programs function effectively because they maintain consistent performance across different testing environments, including clinical and population-based settings (Figure 6).

#### 3.7.6 Group 6: population-based/community screening

**Figure 7:**
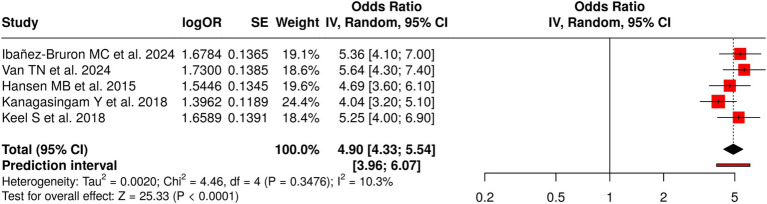
Forest plot of the studies about population-based/community screening.

Group 6 conducted five studies investigating diabetic retinopathy screening methods using AI-powered retinal analysis technology for population-based and community-level assessments. The meta-analysis showed a significant pooled effect using a random-effects model, which applied the inverse-variance method to produce an odds ratio of 4.90, with a 95% confidence interval ranging from 4.33 to 5.54 (*p* < 0.05). The studies produced consistent effect sizes because they showed no significant differences between their results. The evaluation of publication bias revealed two potential problems. The funnel plot showed an asymmetrical pattern, a finding that was statistically confirmed by Egger’s test (intercept = 14.64, 95% CI: 8.47–20.81, t = 4.648, *p* = 0.019). The results demonstrate that community-based diabetic retinopathy screening programs successfully identify cases of diabetic retinopathy across entire populations. The results show potential publication bias, which requires careful assessment, as the findings need validation through additional real-world research across different community environments (Figure 7).

### 3.8 Publication bias

**Figure 8:**
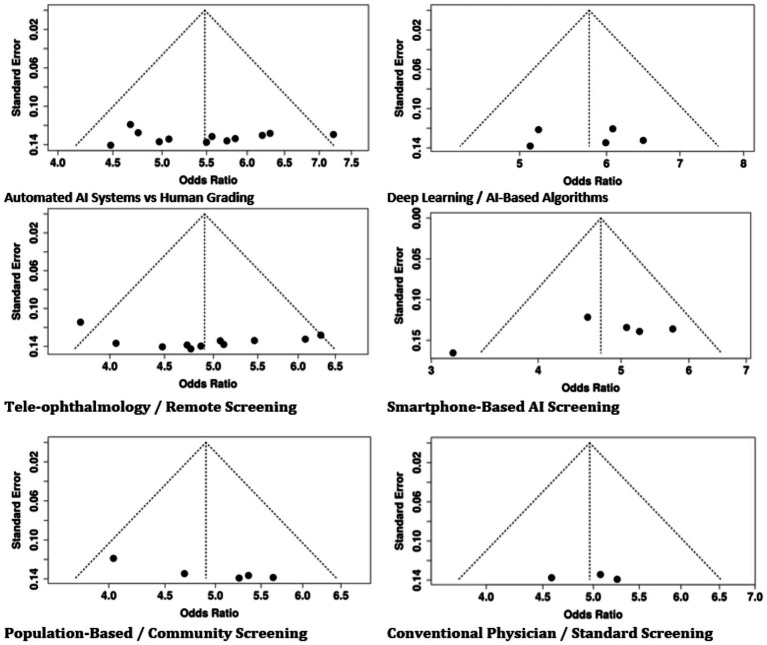
Funnel plot of the included studies.

The analyzed studies on diabetic retinopathy (DR) screening showed minimal publication bias across all three testing methods, including AI-based tele-ophthalmology and standard screening methods. The funnel plot analyses for deep learning/AI-based algorithms and automated AI systems versus human grading, tele-ophthalmology, and conventional physician screening methods yielded symmetrical results, indicating the absence of small-study effects. The results of Egger’s test confirmed this finding, as the study showed non-significant intercepts (*p* > 0.05), indicating minimal risk of reporting bias. The community- and population-based screening studies demonstrated some degree of publication bias, as indicated by Egger’s test, which suggested that smaller studies with negative results were likely missing from this research category. The findings show that most of their results hold when community-based interventions require researchers to interpret data with special care (Figure 8).

## 4 Discussion

The current systematic review and meta-analysis synthesizes evidence from 45 studies evaluating artificial intelligence (AI), tele-ophthalmology, and smartphone- and conventional-based, as well as population-level, screening methods for diabetic retinopathy (DR) testing. The studies demonstrate how emerging technologies can perform DR screening tests, highlighting their practical testing challenges and their ability to integrate into routine medical evaluations. The research results show that AI-based systems provide diagnostic precision that matches or surpasses that of human examiners and can be used in primary care facilities, community settings, and low-resource environments.

Six research studies assessed deep learning (DL) algorithms, yielding an overall summary odds ratio (OR) of 5.79 (95% CI: 5.22–6.42), indicating a strong association between the algorithms and their ability to detect diabetic retinopathy (DR) accurately. The models were trained and validated on extensive datasets that included complete expert ophthalmologist reference standards. The studies used consecutive image inclusion methods, employed blinded AI assessment, and followed rigorous grading protocols, resulting in a low risk of bias. The main strength of DL-based methods lies in their ability to scale via automated systems, reducing the need for expert graders while preserving diagnostic accuracy. The researchers found that the generalizability of their findings was limited because most studies took place in high-income hospitals, which resulted in restricted access to rural areas with limited resources and diverse ethnic groups. The research studies focused on fundus photographs rather than on multimodal imaging or symptomatic indicators, thereby limiting their findings. The system faces performance challenges in real-world settings due to variability in image quality among patient populations.

The research, which analyzed automated AI systems across 13 studies and compared them to human grading, produced the same results, yielding an overall summary OR of 5.48 with a 95% confidence interval of 5.09 to 5.90. The studies demonstrate that their results are replicable, as the researchers found identical patterns across various study groups and locations. The studies demonstrate that clinical workflows can successfully use AI to enable rapid patient triage while reducing the demands placed on ophthalmologists. The study demonstrates three main advantages: its multicenter research design, its use of separate testing datasets, and its complete presentation of diagnostic performance data, including sensitivity, specificity, and predictive values. The research presents two main constraints which limit study participants to either trial sites or hospital sites while failing to include symptomatic factors which affect clinical decisions across different populations.

The five studies that evaluated smartphone-based AI screening showed a lower summary OR of 4.73 (95% CI, 3.96–5.66) and, at the same time, moderate heterogeneity (I^2^ = 54%). The use of smartphone fundus cameras enables users to conduct accessible and affordable eye examinations in remote areas but the technology faces challenges with image quality and operator training and device calibration which result in variable test outcomes. The studies demonstrate that DR screening can be expanded to underserved areas through AI technology that doctors can use on portable devices.

The critical factors that need evaluation in this research include the creation of unified imaging techniques, the development of AI models that can perform on substandard image quality, and the establishment of follow-up systems for all identified cases.

The study results demonstrate that tele-ophthalmology and remote screening methods tested across 11 studies achieved an overall operational result that did not vary from remote image interpretation results, with AI support showing high accuracy. The system provides benefits through its operational efficiency improvements and its ability to operate within primary healthcare systems while enabling ongoing patient monitoring. The organization needs to establish adequate systems, recruit qualified staff, and implement standardized assessment methods to achieve successful outcomes. The five studies demonstrated that community-based screening methods achieved an operational result of 4.90, with a 95% confidence interval of 4.33 to 5.54. The programs show promise for widespread detection of diabetic retinopathy; however, the funnel plot and Egger’s test results indicate publication bias, as studies with positive results are more likely to be published. The researchers needed to implement advanced research design methods alongside comprehensive reporting techniques to avoid overstating AI’s actual capabilities in population studies. The research studies lost their external validity because of their regional focus which excluded specific demographic groups from study.

The QUADAS-2 assessment showed that most studies had a low risk of bias. This was because they carefully selected patients, used index tests, and applied reference standards, all while following proper procedures and timelines. Several studies present unclear risk estimates because they lack complete reporting, particularly affecting multi-disease datasets and conference reports that fail to disclose their patient sampling methods or criteria for consecutive image inclusion. The AI-based DR research field requires standardized reporting guidelines that enable researchers to present their datasets, grading protocols, and symptomatic indicators in a way that supports external validity. The combination of artificial intelligence and tele-ophthalmology provides healthcare professionals with powerful tools that enhance diabetic retinopathy screening by improving efficiency, enabling earlier diagnosis, and expanding access to testing for remote communities. Deep learning algorithms and autonomous artificial intelligence systems achieve high sensitivity and specificity, enabling them to reduce diagnostic errors and enhance timely patient referral. The public can access services through smartphone platforms and community programs which require proper management of image quality and operator education and follow-up procedures.

The evidence demonstrates that AI-based tele-ophthalmology screening systems for DR detection perform well across diverse environments because deep learning algorithms and automated grading systems achieve the highest diagnostic accuracy. The population-level screening methods that use smartphones as their basis offer practical options for screening patients, although their results show noticeable differences. The existing research limitations, which affect generalizability and the absence of symptomatic data and specific device performance data, require researchers to conduct studies that validate results across both specific contexts and real-world applications. The research should investigate community-based, multiethnic groups undergoing multimodal imaging tests that incorporate AI systems into clinical operations to maximize the benefits of DR screening for public health.

## 5 Conclusion

Artificial intelligence tele-ophthalmology smartphone-based systems and traditional screening methods all show strong capability to identify diabetic retinopathy in patients while AI systems deliver superior accuracy in their diagnostic results. Deep learning combined with automated AI systems provides dependable solutions which enable healthcare facilities to minimize their need for specialized graders while increasing their ability to conduct screenings especially in primary care clinics and remote regions. The population-based screening methods which use smartphones as a base create practical solutions for screening patients; however, organizations should establish methods to handle differences in image resolution and particular environmental elements. The integration of AI technology into diabetic retinopathy screening programs enables earlier detection, more efficient clinic operations, and better patient outcomes. However, the technology needs to be tested in diverse real-world settings to confirm its effectiveness.

## Data Availability

The raw data supporting the conclusions of this article will be made available by the authors, without undue reservation.
